# Three-dimensional assessment of teeth first-, second- and third-order position in Caucasian and African subjects with ideal occlusion

**DOI:** 10.1186/s40510-015-0086-9

**Published:** 2015-05-26

**Authors:** Luca Lombardo, Alessandro Perri, Angela Arreghini, Michele Latini, Giuseppe Siciliani

**Affiliations:** Department of Orthodontics, University of Ferrara, Via montebello, 31, Ferrara, 44100 Italy

**Keywords:** Tip, Torque, In-out, Straight-wire prescription, Andrews

## Abstract

**Background:**

The aim of this study was to provide an updated version of Andrews’ seminal study by exploiting 3D software to analyse the tip, torque and in-out values of two groups of different racial and ethnic background.

**Methods:**

The analysis was conducted on one Caucasian group (30 individuals) and one of African origin (29). All subjects were adult, in normal occlusion and had no previous history of orthodontic treatment. Rhinoceros™ 3D modelling software was used to identify anatomical reference points, planes and axes and to make the appropriate measurements.

**Results:**

Compared to Andrews’ measurements, we found more positive coronal tip values in both African and Caucasian subjects, while the torque values we measured tended to be less negative in the posterior sectors than those reported by Andrews. We measured greater tip values in the lower jaw of Caucasian with respect to African subjects, particularly in the middle sectors.

**Conclusions:**

Race and ethnicity have a strong influence on values of tip, torque and in-out. This is translated as a more positive tip in Caucasian subjects and a more positive torque in those of African descent (greater proclination of the incisors). Finally, with respect to the values reported by Andrews, we found a tendency to more positive mean tip (except for at the upper second molars and lower incisors) and less negative torque in the posterior sectors.

## Background

Andrews was the first to put forward the hypothesis that the ideal occlusion of untreated patients could serve as the target of orthodontic treatment [1]. However, despite Andrews’ undeniable influence on orthodontics as we know it today, several authors [1–6] have identified sources of bias in this seminal study, in both the method Andrews adopted for making his measurements—ascribable to the instruments he used—and the sample he selected—North American subjects of Caucasian origin. Nevertheless, only Sebata [2], Watanabe [3], Currim [4] and Doodamani [5] have since replicated Andrews’ study using different samples (Japanese in the first two, Indians in the latter two) and a modified method. As could be expected from the different approaches used and samples considered in these investigations, all four produced results that were significantly different from those of Andrews.

In spite of the many advances in orthodontics since Andrews’ proposed his pre-programmed appliance, the majority of devices on the market still feature prescriptions based on his measurements. This means that, generally speaking, not enough attention is paid to the position of the roots. In reality, there is a strong correlation between the tip and torque and the tooth root position, as there are variations in coronal morphology, incongruencies between the inclination of the roots and the crown, and a disproportion between the height of the crown and the length of the root of the same tooth [7].

That being said, measurement of the angulation and inclination of the roots relies on 3D imaging, which has only been available in recent years following the development of such diagnostic tool technology. In this regard, the study by Tong [8] was a real innovation; its aim was to analyse the tip and torque of the teeth in patients in normal, or nearly normal, occlusion by means of CBCT. The only flaw in that study was the fact that it was carried out on a small sample of single ethnicity (13 Caucasians).

Huanca Ghislanzoni et al. [9] have validated a method for the analysis of 3D virtual casts, which allow to identify the values of the first, second and third order of teeth with great intra- and inter-operator reproducibility. This confirms the potential of new technologies in obtaining reliable data for clinical diagnosis and the tooth position.

As mentioned, to date, no researcher has attempted to replicate Andrews’ work exploiting the potential of today’s technology, which provides a far greater degree of accuracy and reproducibility than can be obtained by the manual methods available in his time. Hence, we set out to compare the values manually measured by Andrews with those, based on the same anatomical reference points and planes, obtained using digital technology to determine whether Andrews’ values are still relevant or whether significant differences indicate that digital measurements provide us with better reference values. We also extended the study to two ethnic groups.

## Methods

### Sample selection

The study was conducted on a sample made up of two groups of adults in ideal occlusion with no previous history of orthodontic treatment, one of Caucasian and one of African origin. Thirty Caucasian subjects (14 males and 16 females) were recruited from among patients presenting for general dentistry procedures or routine check-up at various private practices in Italy, and 29 African subjects (14 males and 15 females) were recruited from among the students of the Eduardo Mondlane University in Inhambane, Mozambique. Subject selection was performed according to the inclusion criteria noted in Table 1.

**Table 1:** Inclusion criteria

Adult age (not less than 18 years)	No previous orthodontic treatment
Regular arch form with little or no crowding	Complete dentition to second molars
No bridges or implants	Centred midlines
No gingival recession	No joint or muscle pathologies
No ectopic teeth	No supernumerary teeth or tooth agenesis
At least four of Andrews’ six keys with bilateral molar and canine class I occlusion always present	Presence of minimal diastems and/or small premolar rotation or little irregularity (Little’s index less than 3) at incisors in some individuals
Overbite and overjet within normal limits 2 mm ± 1 mm	No anterior or posterior cross-bite
No markedly visible intra-oral or extra-oral symmetry	

**Fig. 1:**
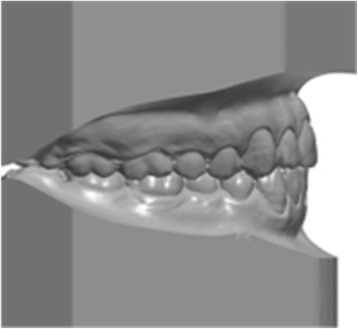
Three-dimensional model

Precision impressions of the dental arcades of each patient were taken using the dual-phase putty and light body (Elite HD+ Fast Set, Zhermack, Rovigo, Italy) technique. Bite registrations of each patient’s dentition were taken in maximum intercuspidation. Silicone was chosen as the impression material due to its precision and dimensional stability [10]. Each subject’s impressions were placed in an orthodontic 3D scanner (3Shape D700/710, Copenhagen, Denmark) to obtain virtual 3D renderings in stereolithography (STL) format (Fig. 1). The anatomical reference points, axes and planes on these renderings were marked using Rhinoceros™ 4.0 3D modelling software (Robert McNeel & Associates, Seattle, USA), which was also used to make the measurements detailed below.

### Measurements taken

**Fig. 2:**
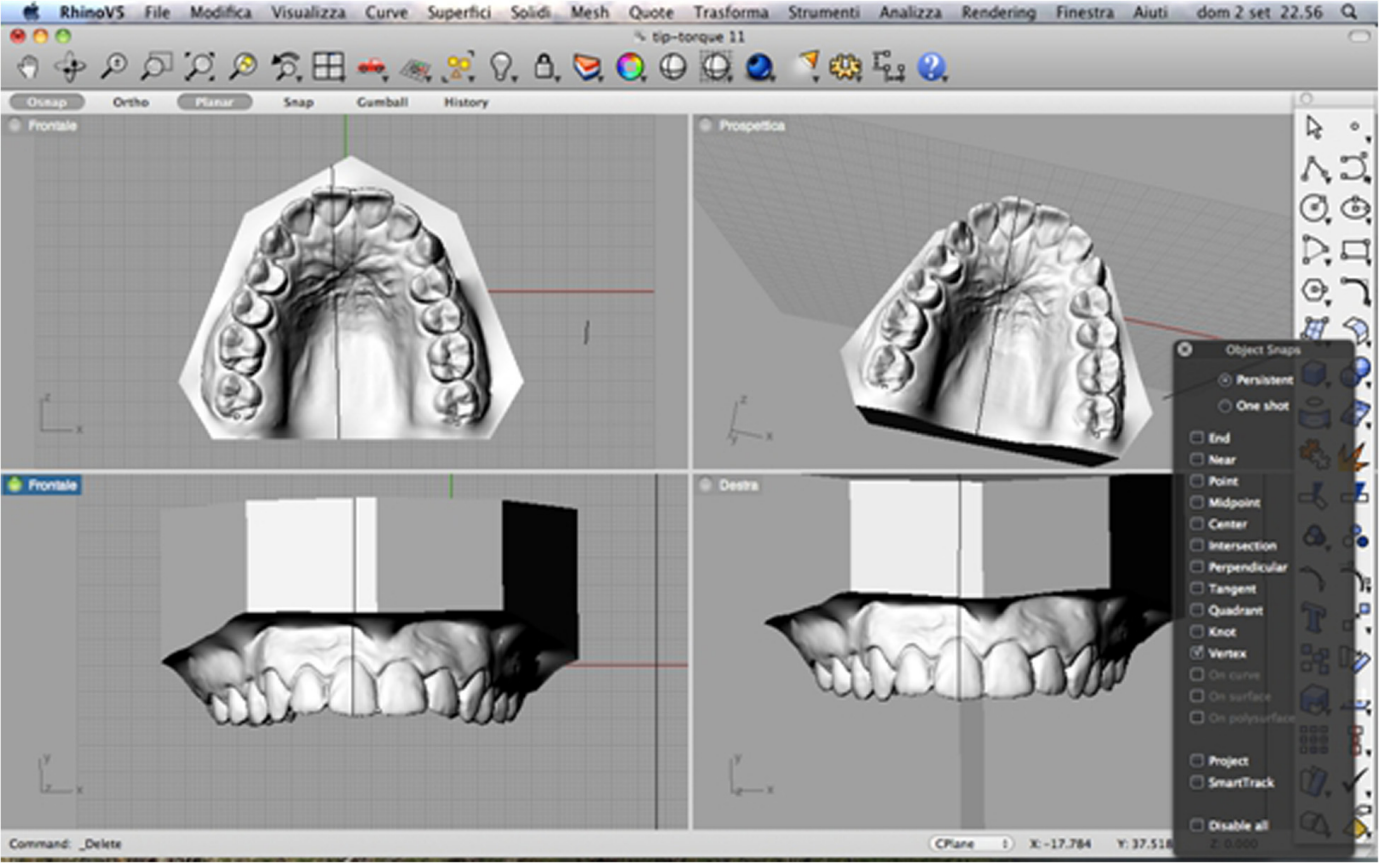
Measuring the FACC

**Fig. 3:**
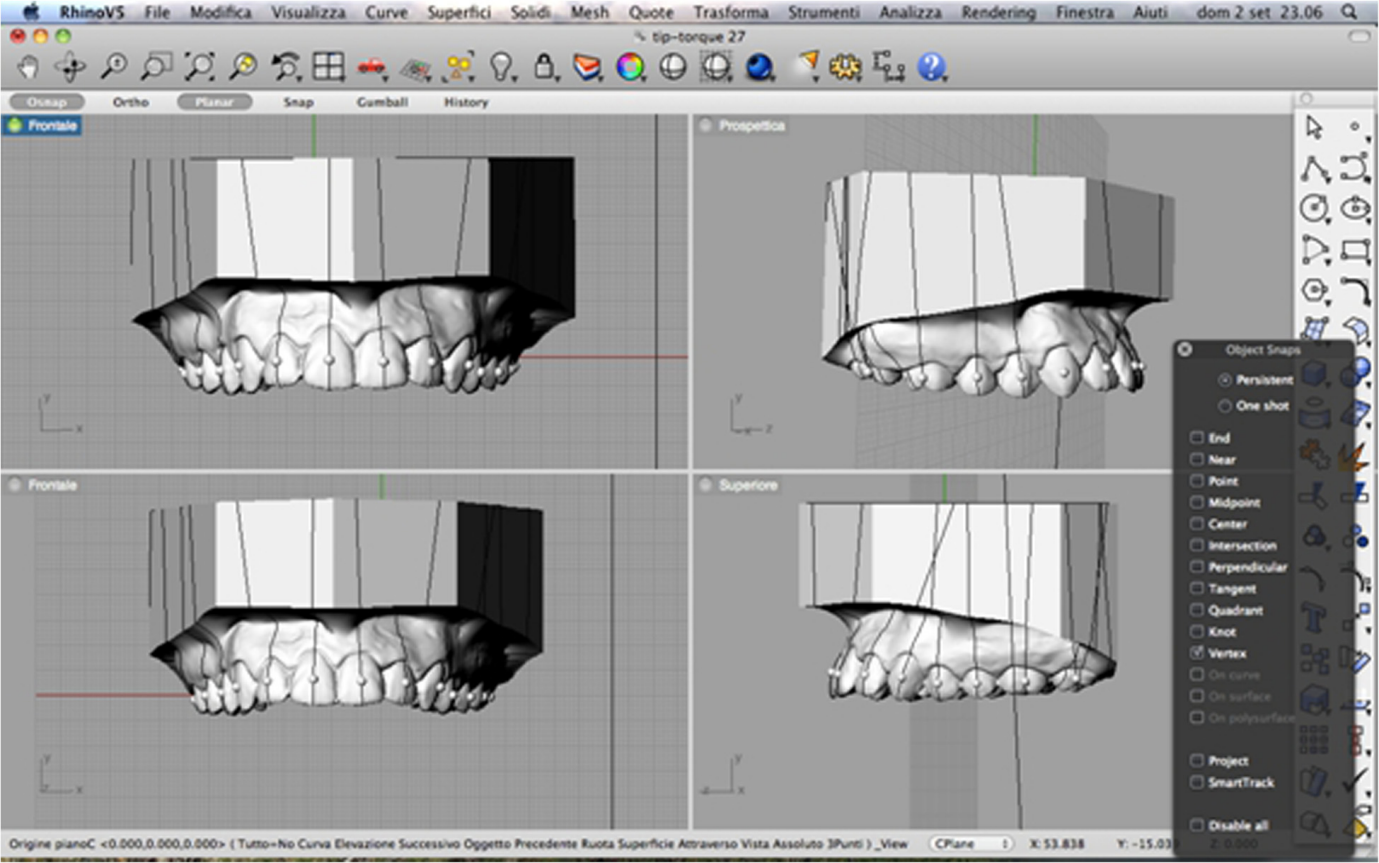
Creation of virtual spheres around FA points

**Fig. 4:**
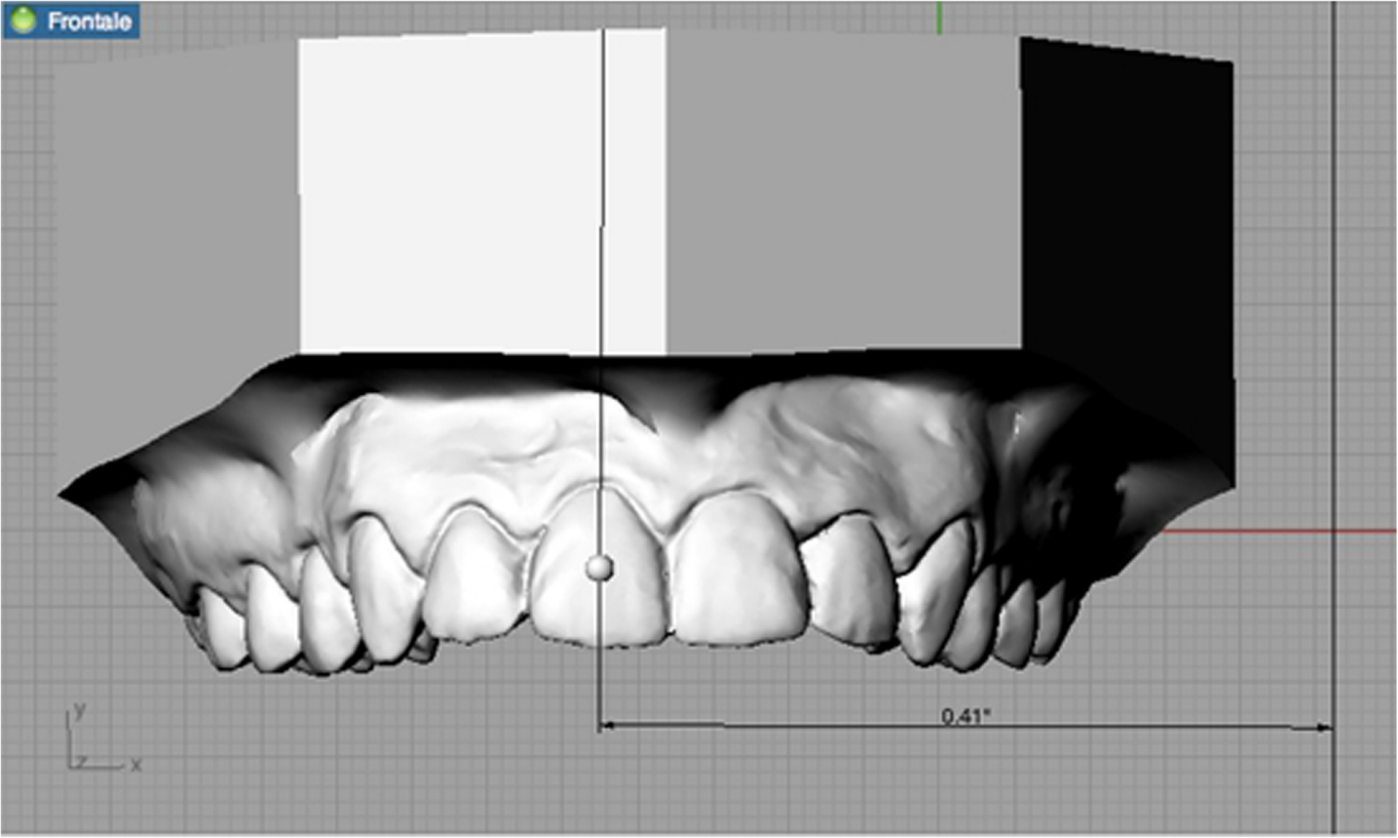
Calculating the upper central incisor tip

**Fig. 5:**
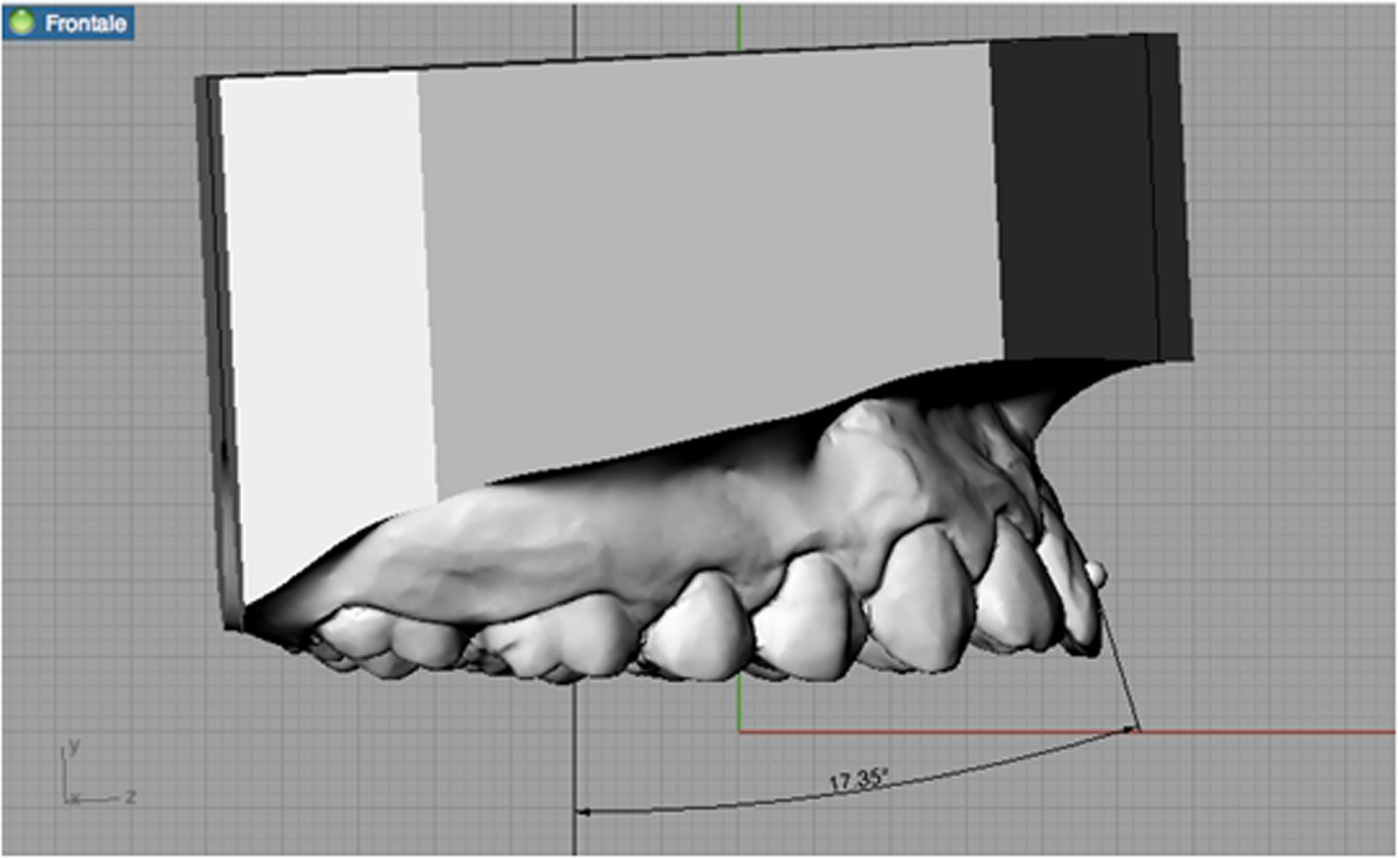
Calculating the upper central incisor torque

Tip and torque: two operators performed measurements. Both operators used Rhinoceros™ software to make the measurements, which were based on the anatomical reference points described by Andrews. The occlusal plane was identified on each 3D rendering and used as a plane of reference. This plane, described by Rhinoceros as the “C-plane”, was made to pass through three anatomical points: the inter-incisal point and the halfway points on the distal marginal crest of each second molar. After the occlusal plane was aligned visually, the line perpendicular to it was traced. The facial axis of each tooth crown (FACC) was marked using the “section” tool, in all cases making sure that the C-plane was aligned correctly. To make the facial axis (FA) point visible, this was placed in the centre of a virtual 1-mm sphere whose centre lay on the previously traced axis (Figs. 2 and 3). Once these anatomical reference points had been marked on the renderings, the tip and torque of each tooth were measured. The tip was taken as the angle between the FACC and the line perpendicular to the occlusal plane, using the “Evaluate/angle” function (Fig. 4). To calculate the torque, a tangent line was drawn perpendicular to the surface of each tooth, passing through the FA point (i.e. the centre of the virtual sphere) on a mesiodistal view of each tooth (therefore positioned at 90° with respect to the FACC), the angle between this line and the occlusal reference plane was calculated (Fig. 5).

**Fig. 6:**
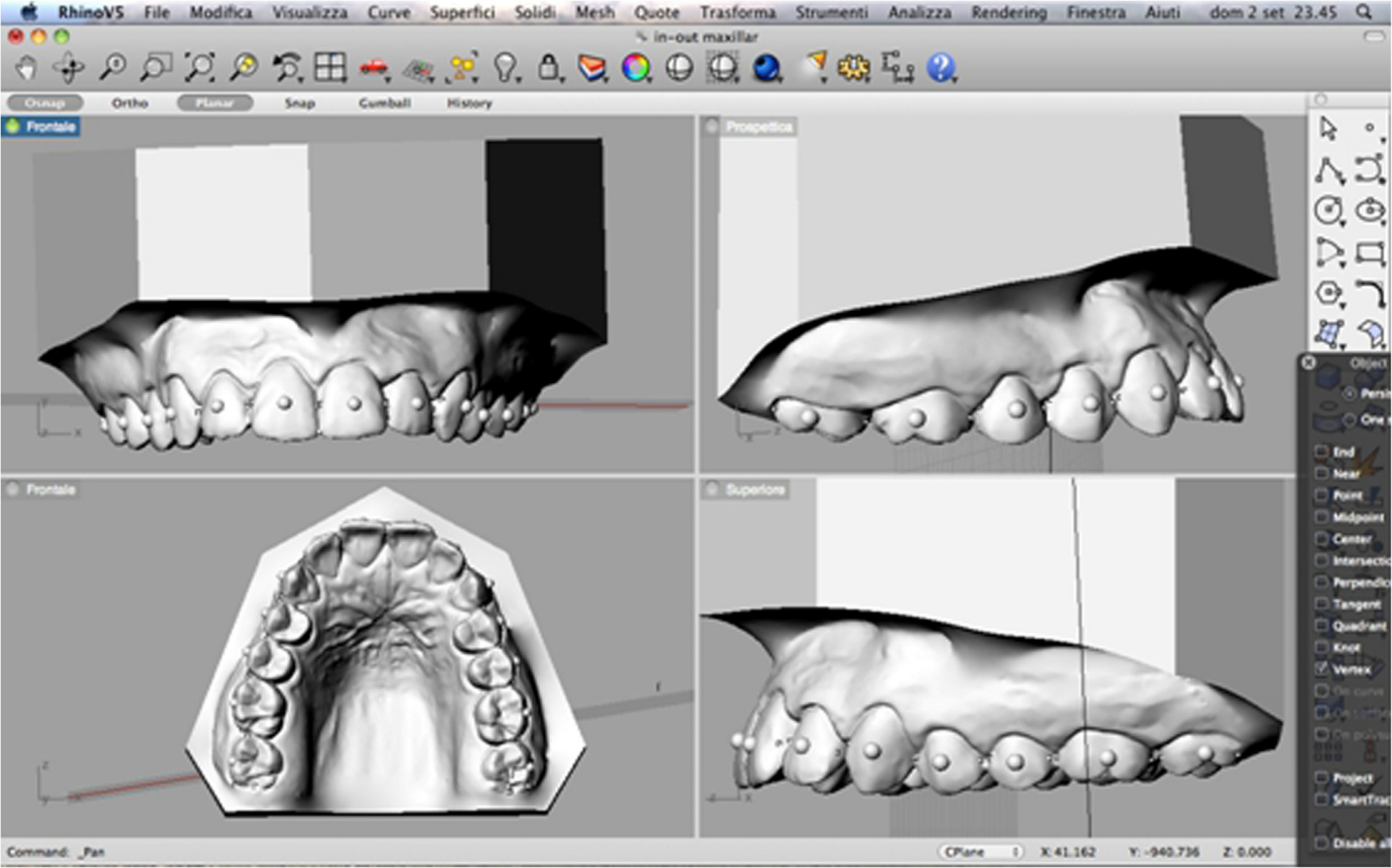
Determining contact points

**Fig. 7:**
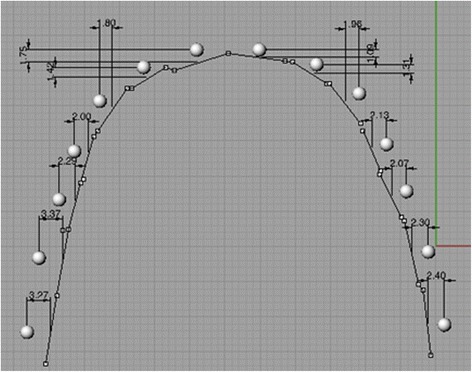
Calculating the in-out

In-out: a plane parallel to the occlusal plane, passing through the FA point of each tooth, was identified, and the mesial, the distal and the most vestibular point of every tooth was selected (Fig. 6). These points were joined together by a line whose perpendicular distance from the FA point (i.e. the centre of the virtual sphere) was taken as the in-out value of that crown. These values were calculated using the ‘“Evaluate/Distance” ! ?/“Evaluate/Length” ! ?’ software tool (Fig. 7).

### Statistical analysis

The mean, standard deviation and standard error of each value obtained above were calculated. The Shapiro-Wilk test was performed to assess the normal distribution of the studied samples. Student’s *t* test for independent samples (significance level *p* < 0.05) was used to compare the group mean values for every tooth. To verify the repeatability of the measurements performed, 10 renderings of the upper arcade and 10 of the lower were selected at random from each ethnic sample. The measurements were then repeated by the other operator, and the second set of measurements was compared with the first by means of measurement systems analysis (MSA) conducted using the *t* test for paired data.

## Results

### African sample

The measurements taken of the upper jaw highlight positive tip values for all the teeth except for the second molars (Table 2). The tip values in the lower jaw tended to be more positive in an anteroposterior direction (Table 2), whereas the torque on both the upper and lower teeth tended to get more negative in an anteroposterior direction (Table 3); only the central and lateral incisors displayed positive torque.

**Table 2:** Comparison of tip values measured for the two groups

Africans—tip				Caucasians—tip			Comparison	
	*n*	Mean	Standard deviation	*n*	Mean	Standard deviation	*t* test	*p* level
TIP U7	58	−3.06	6.78	60	−3.88	10.60	0.51	ns
TIP U6	58	9.48	3.53	60	10.26	5.54	−0.98	ns
TIP U5	58	5.96	5.84	60	9.64	4.16	−3.93	<0.001
TIP U4	58	3.29	4.45	60	7.67	4.28	−5.45	<0.001
TIP U3	58	8.23	5.20	60	9.96	4.81	−1.87	ns
TIP U2	58	9.23	3.87	60	9.99	3.69	−1.10	ns
TIP U1	58	3.68	3.55	60	4.53	2.84	−1.44	ns
TIP L7	58	12.65	5.83	60	14.20	6.47	−1.37	ns
TIP L6	58	6.30	5.36	60	10.99	2.78	−5.95	<0.001
TIP L5	58	3.60	4.02	60	6.90	3.09	−4.99	<0.001
TIP L4	58	2.95	3.89	60	6.06	3.36	−4.63	<0.001
TIP L3	58	3.478	4.850	60	5.91	3.98	−2.98	0.004
TIP L2	58	−0.26	3.01	60	0.14	4.46	−0.57	ns
TIP L1	58	−1.13	3.30	60	0.00	4.46	−1.57	ns

*ns* not significant.

**Table 3:** Comparison of torque values measured for the two groups

Africans—torque				Caucasians—torque			Comparison	
	*n*	Mean	Standard deviation	*n*	Mean	Standard deviation	*t* test	*p* level
TORQUE U7	58	−12.79	10.11	60	−5.50	12.23	−3.54	<0.001
TORQUE U6	58	−10.98	9.75	60	−6.26	10.24	−2.56	0.013
TORQUE U5	58	−4.00	8.43	60	−3.54	6.42	−0.33	ns
TORQUE U4	58	−4.49	8.38	60	−5.35	5.64	0.65	ns
TORQUE U3	58	−2.03	7.95	60	−3.35	7.15	0.95	ns
TORQUE U2	58	7.41	8.14	60	6.23	6.31	0.88	ns
TORQUE U1	58	11.41	6.28	60	7.41	6.19	3.49	<0.001
TORQUE L7	58	−32.51	10.93	60	−33.26	11.69	0.36	ns
TORQUE L6	58	−23.50	9.57	60	−29.24	9.29	3.31	0.003
TORQUE L5	58	−11.39	8.52	60	−17.43	7.98	3.97	<0.001
TORQUE L4	58	−9.23	7.02	60	−14.96	7.69	4.23	<0.001
TORQUE L3	58	−1.95	7.49	60	−9.01	5.74	5.73	<0.001
TORQUE L2	58	5.50	8.26	60	−1.36	6.89	4.90	<0.001
TORQUE L1	58	9.68	9.18	60	2.19	7.11	4.94	<0.001

*ns* not significant.

### Caucasian sample

With the exception of the second molar, tip values in the upper jaw were all positive, displaying a tendency to decrease from the anterior to the posterior sectors (Table 2). The lower tip values were all positive, but tended to increase in an anteroposterior direction (Table 2). As for torque, in the upper jaw, this showed a tendency to increase in negativity in an anteroposterior direction, being positive at the incisors and negative in the canines, premolars and molars (Table 3). In the lower jaw, increasingly negative torque values were measured in an anteroposterior direction. Only the central incisors had a positive torque while the most negative torque was seen at the second molars (Table 3).

### Measurement system analysis

From the analysis of the measurement method, in results that there are no statistically significant differences (significance (two-tailed) <0.05) between the values of tip and torque measured by the two operators, both in the Caucasian and the African groups.

### Comparison of tip and torque of African and Caucasian groups

A comparison of the mean tip and torque values obtained for each ethnic sample was performed using Student’s *t* test for independent samples. This showed that, in terms of tip values in the upper teeth, there were only significant differences between the two groups at the premolars, whose mean tip in the Caucasian samples was roughly 4° more positive than that in the African group. In the lower jaw, comparable values were only found at the incisors, whereas the tip at the canines, premolars and molars was significantly more positive in the Caucasian sample, at 2.5°, 3° and 4° greater, respectively (Table 2).

**Fig. 8:**
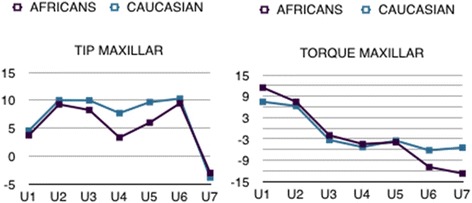
Comparing African and Caucasian tip and torque values for the upper jaw

**Fig. 9:**
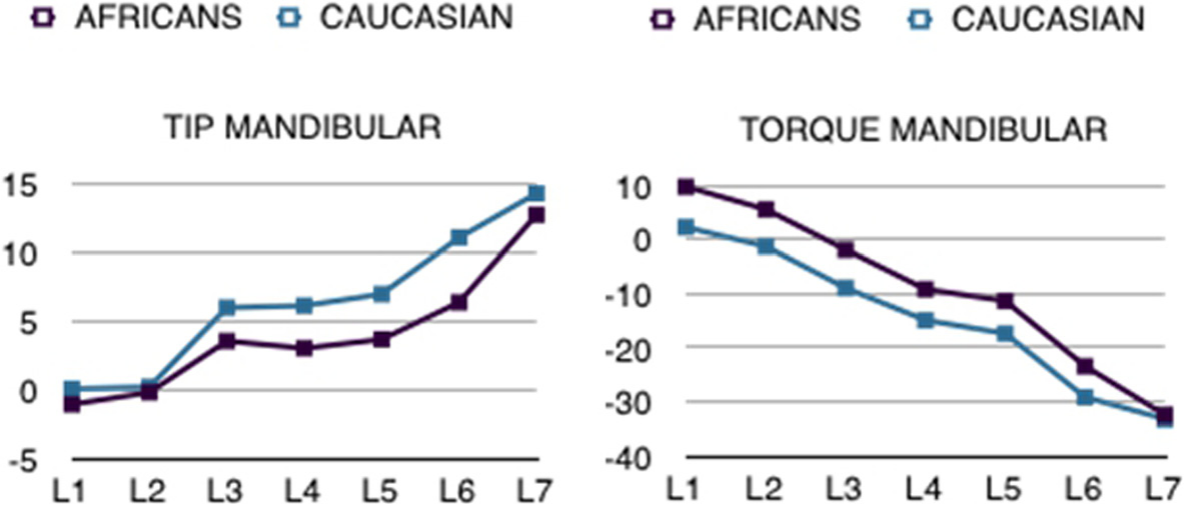
Comparing African and Caucasian tip and torque values for the lower jaw

Statistically significant differences were also found in the torque values measured for the upper arch, this time at the central incisors and both sets of molars. In each case, absolute torque values were greater in the African subjects, with a torque 4° more positive at the central incisors and roughly 5° and 7°, respectively, at the first and second molars (Table 3). Among the lower teeth, only the second molar torque of the two groups was comparable; all the other teeth displayed more positive torque in the African group, reaching statistical significance in the canines, premolars and first molars. In particular, the torque on the central and lateral incisors was, respectively, roughly 7° and 6° more positive in the African sample. Analogously, the canines displayed roughly 7° less negative torque in the African sample, and approximately 6° less negative torque was measured at the premolars and first molars (Table 3) (Figs. 8 and 9).

### Comparison of tip and torque of African and Caucasian groups with values reported by Andrews

Statistically significant differences with respect to values reported by Andrews were found in terms of the tip values measured in the upper jaw of both African and Caucasian subjects [10] (Tables 4 and 5). In the African sample, only the tip measured at the upper central incisors, canines and first premolars was comparable with values reported by Andrews (differences of <1°) (Table 4). In the Caucasian subjects, all upper crowns had angulation values significantly different to those measured in Andrews’ original sample of North American subjects. The greatest differences were seen at the molars and premolars, and in our Caucasian sample, only the second molars had a negative tip (Table 5). A similar discrepancy was found in the lower jaw (Table 4); in the African group, substantial differences were noted at the lower incisors, which presented negative tip, at the first and second premolars and in particular at the first and second molars, which both had considerably greater angulation values in our sample, the difference being as much as 10° at the second molars. Likewise, in our Caucasian group, lower tip values were markedly different to those reported by Andrews, especially in the posterior sectors, in which the crowns displayed a more positive tip (Table 5).

**Table 4:** Comparison of our African tip values with those measured by Andrews

Africans—tip				Andrews’ values			Comparison	
	*n*	Mean	Standard deviation	*n*	Mean	Standard deviation	*t* test	*p* level
TIP U7	58	−3.06	6.78	240	.39	5.69	−3.58	<0.001
TIP U6	58	9.43	3.53	240	5.73	1.90	7.71	<0.001
TIP U5	58	5.96	5.84	240	2.82	1.52	4.07	<0.001
TIP U4	58	3.29	4.45	240	2.65	1.69	1.07	ns
TIP U3	58	8.23	5.20	240	8.40	2.97	−.24	ns
TIP U2	58	9.23	3.87	240	8.04	2.80	2.20	0.031
TIP U1	58	3.68	3.55	240	3.59	1.65	.18	ns
TIP L7	58	12.65	5.83	240	2.94	2.05	12.50	<0.001
TIP L6	58	6.30	5.36	240	2.03	1.14	6.03	<0.001
TIP L5	58	3.60	4.02	240	1.54	1.35	3.84	<0.001
TIP L4	58	2.95	3.89	240	1.28	1.90	3.19	0.002
TIP L3	58	3.478	4.850	240	2.48	3.28	1.49	ns
TIP L2	58	−.26	3.01	240	.38	1.47	−1.57	ns
TIP L1	58	−1.13	3.30	240	.53	1.29	−3.77	<0.001

*ns* not significant.

**Table 5:** Comparison of our Caucasian tip values with those measured by Andrews

Caucasians—tip				Andrews’ values			Comparison	
	*n*	Mean	Standard deviation	*n*	Mean	Standard deviation	*t* test	*p* level
TIP U7	60	−3.88	10.60	240	.39	5.69	−3.02	0.004
TIP U6	60	10.26	5.54	240	5.73	1.90	6.24	<0.001
TIP U5	60	9.64	4.16	240	2.82	1.52	12.49	<0.001
TIP U4	60	7.67	4.28	240	2.65	1.69	8.91	<0.001
TIP U3	60	9.96	4.81	240	8.40	2.97	2.40	0.019
TIP U2	60	9.99	3.69	240	8.04	2.80	3.83	<0.001
TIP U1	60	4.53	2.84	240	3.59	1.65	2.46	0.017
TIP L7	60	14.20	6.47	240	2.94	2.05	13.32	<0.001
TIP L6	60	10.99	2.78	240	2.03	1.14	24.47	<0.001
TIP L5	60	6.90	3.09	240	1.54	1.35	13.12	<0.001
TIP L4	60	6.06	3.36	240	1.28	1.90	10.60	<0.001
TIP L3	60	5.913	3.980	240	2.48	3.28	6.18	<0.001
TIP L2	60	.14	4.46	240	.38	1.47	−.41	ns
TIP L1	60	.00	4.46	240	.53	1.29	−.90	ns

*ns* not significant.

As regards the torque in the upper arcade, we found a greater positive tendency in the African subjects with respect to Andrews’ measurements. This was true of all teeth except for the second molars, which had a more negative inclination in our sample (Table 6). The only upper teeth in which there were no statistically significant differences in the Caucasian group were the central incisors and second molars; the torque on the upper lateral incisors and first premolars was roughly 2–3° lower, and this discrepancy reached 4–5° at the canines, second premolars and first molars (Table 7). In the lower jaw, the torque values we measured in African subjects were in all cases more positive than those reported by Andrews. Differences were statistically significant in all cases, from a minimum of roughly 5° at the second molar, up to approximately 12° at the second premolar (Table 6). In the Caucasians we studied, the torque on the lower molars and lateral incisors was comparable to Andrews’ measurements, while the torque we measured at the two premolars was less negative. In contrast to the other teeth, the central incisors displayed a positive torque (Table 7).

**Table 6:** Comparison of our African torque values with those measured by Andrews

Africans—torque				Andrews’ values			Comparison	
	*n*	Mean	Standard deviation	*n*	Mean	Standard deviation	*t* test	*p* level
TORQUE U7	58	−12.79	10.11	240	−8.10	5.63	−3.41	0.001
TORQUE U6	58	−10.98	9.75	240	−11.53	3.91	.42	ns
TORQUE U5	58	−4.00	8.43	240	−8.78	4.13	4.20	<0.001
TORQUE U4	58	−4.49	8.38	240	−8.47	4.02	3.52	<0.001
TORQUE U3	58	−2.03	7.95	240	−7.25	4.21	4.84	<0.001
TORQUE U2	58	7.41	8.14	240	4.42	4.38	2.70	0.009
TORQUE U1	58	11.41	6.28	240	6.11	3.97	6.14	<0.001
TORQUE L7	58	−32.51	10.93	240	−36.03	6.57	2.35	0.030
TORQUE L6	58	−23.50	9.57	240	−30.67	5.90	5.46	<0.001
TORQUE L5	58	−11.39	8.52	240	−23.63	5.58	10.41	<0.001
TORQUE L4	58	−9.23	7.02	240	−18.95	4.96	9.96	<0.001
TORQUE L3	58	−1.95	7.49	240	−12.73	4.65	10.48	<0.001
TORQUE L2	58	5.50	8.26	240	−3.24	5.37	7.68	<0.001
TORQUE L1	58	9.68	9.18	240	−1.71	5.79	9.02	<0.001

*ns* not significant

**Table 7:** Comparison of our Caucasian torque values with those measured by Andrews

Caucasians—torque				Andrews’ values			Comparison	
	*n*.	Mean	Standard deviation	*n*	Mean	Standard deviation	*t* test	*p* level
TORQUE U7	60	−5.50	12.23	240	−8.10	5.63	1.60	ns
TORQUE U6	60	−6.26	10.24	240	−11.53	3.91	3.92	<0.001
TORQUE U5	60	−3.54	6.42	240	−8.78	4.13	6.02	<0.001
TORQUE U4	60	−5.35	5.64	240	−8.47	4.02	4.04	<0.001
TORQUE U3	60	−3.35	7.15	240	−7.25	4.21	4.05	<0.001
TORQUE U2	60	6.23	6.31	240	4.42	4.38	2.10	0.040
TORQUE U1	60	7.41	6.19	240	6.11	3.97	1.54	ns
TORQUE L7	60	−33.26	11.69	240	−36.03	6.57	1.77	ns
TORQUE L6	60	−29.24	9.29	240	−30.67	5.90	1.14	ns
TORQUE L5	60	−17.43	7.98	240	−23.63	5.58	5.68	<0.001
TORQUE L4	60	−14.96	7.69	240	−18.95	4.96	3.83	<0.001
TORQUE L3	60	−9.01	5.74	240	−12.73	4.65	4.66	<0.001
TORQUE L2	60	−1.36	6.89	240	−3.24	5.37	1.97	ns
TORQUE L1	60	2.19	7.11	240	−1.71	5.79	3.93	<0.001

*ns* not significant.

### Analysis of in-out values

In both jaws of our African sample (Table 8), there is an observable tendency for the in-out values to increase progressively from the lateral incisor to the second molar. The same was true for the Caucasian subjects we measured (Table 8). However, a comparison of our ethnic groups revealed that the in-out values were greater in all teeth of the African upper jaw except for the second molars (Table 8). In the lower jaw, we found statistically significant differences at both sets of incisors and the second premolars, whose torque values were greater in the African group. In contrast, the second molar had a greater prominence, roughly 0.6 mm (statistically significant), in our Italian Caucasian sample (Table 8).

**Table 8:** Comparison of African and Caucasian in-out values

Africans—in-out				Caucasians—in-out			Comparison	
	*n*	Mean	Standard deviation	*n*	Mean	Standard deviation	*t* test	*p* level
U1	58	1.62	.30	58	1.48	.30	2.60	0.012
U2	58	1.25	.24	58	1.15	.24	2.11	0.039
U3	58	1.65	.31	58	1.66	.34	−.30	ns
U4	58	1.89	.33	58	1.61	.24	5.20	<0.001
U5	58	1.85	.29	58	1.66	.29	3.60	<0.001
U6	58	2.29	.40	58	2.01	.47	3.35	0.001
U7	58	2.47	.56	58	2.75	.62	−2.51	0.015
L1	58	1.18	.21	58	1.01	.32	3.42	0.001
L2	57	1.16	.19	58	.93	.32	4.65	<0.001
L3	58	1.36	.28	58	1.38	.27	−.41	ns
L4	58	2.01	.38	58	1.93	.28	1.30	ns
L5	56	2.06	.45	58	1.91	.32	2.06	0.044
L6	58	2.53	.37	58	2.66	.38	−1.96	ns
L7	56	2.22	.65	58	2.83	.72	−4.76	<0.001

*ns* not significant.

Both of our samples were significantly different from that of Andrews in terms of in-out. In both of our samples, the mean prominence was greater than in Andrews’ North American group at all teeth (Tables 9 and 10).

**Table 9:** Comparison of our African in-out values with those measured by Andrews

Africans—in-out				Andrews—in out			Comparison	
	*n*	Mean	Standard deviation	*n*	Mean	Standard deviation	*t* test	*p* level
U1	58	1.62	.30	240	2.01	.32	−8.73	<0.001
U2	58	1.25	.24	240	1.84	.30	−15.82	<0.001
U3	58	1.65	.31	240	2.67	.39	−21.40	<0.001
U4	58	1.89	.33	240	2.54	.35	−13.36	<0.001
U5	58	1.85	.29	240	2.48	.36	−14.22	<0.001
U6	58	2.29	.40	240	2.88	.40	−10.17	<0.001
U7	58	2.47	.56	238	3.00	.51	−6.58	<0.001
L1	58	1.18	.21	240	1.59	.27	−12.61	<0.001
L2	58	1.16	.19	240	1.64	.32	−14.63	<0.001
L3	58	1.36	.28	240	2.37	.40	−22.57	<0.001
L4	58	2.01	.38	240	2.72	.43	−12.52	<0.001
L5	56	2.06	.45	240	2.60	.34	−8.49	<0.001
L6	58	2.53	.37	240	3.02	.40	−9.00	<0.001
L7	58	2.22	.65	236	2.79	.47	−6.22	<0.001

**Table 10:** Comparison of our Caucasian in-out values with those measured by Andrews

Caucasians—in-out				Andrews—in-out			Comparison	
	*n*	Mean	Standard deviation	*n*	Mean	Standard deviation	*t* test	*p* level
U1	60	1.48	0.30	240	2.01	0.32	−11.88	<0.001
U2	60	1.15	0.24	240	1.84	0.30	−18.36	<0.001
U3	60	1.66	0.34	240	2.67	0.39	−19.75	<0.001
U4	60	1.61	0.24	240	2.54	0.35	−24.19	<0.001
U5	60	1.66	0.29	240	2.48	0.36	−18.53	<0.001
U6	60	2.01	0.47	240	2.88	0.40	−12.86	<0.001
U7	60	2.75	0.62	238	3.00	0.51	−2.85	0.006
L1	60	1.01	0.32	240	1.59	0.27	−12.97	<0.001
L2	60	0.93	0.32	240	1.64	0.32	−14.98	<0.001
L3	60	1.38	0.27	240	2.37	0.40	−22.91	<0.001
L4	60	1.93	0.28	240	2.72	0.43	−17.36	<0.001
L5	60	1.91	0.32	240	2.60	0.34	−14.55	<0.001
L6	60	2.66	0.38	240	3.02	0.40	−6.37	<0.001
L7	60	2.83	0.72	236	2.79	0.47	0.37	ns

*ns* not significant.

## Discussion

On the whole, our findings from this part of the study are in line with those previously reported in the literature [2–5, 8], namely that ethnic background appears to play a significant role in determining the angulation and inclination of the teeth in both arcades. In certain circumstances, these ethnic differences can be great enough (i.e. greater than 3°) to take on clinical relevance.

Indeed, although our sample size was relatively small, if our measurements were taken as reference values, orthodontic bracket prescription would need to be altered or bends made in the wire to obtain optimal finishing in the Mozambican population.

When comparing the values we measured in our Italian sample with those reported by Andrews, it became clear that on the whole, their teeth had a more positive tip (i.e. a greater mesiodistal angulation). Similarly, torque values were larger on the whole in our sample. Hence, the results of this part of the study also agree with the literature, showing that individuals of the same race have different tip and torque values, depending on their ethnic background. Indeed, a similar picture was seen in two different Japanese [2] and Indian [4] samples, as well as Andrews’ North American Caucasians.

**Fig. 10:**
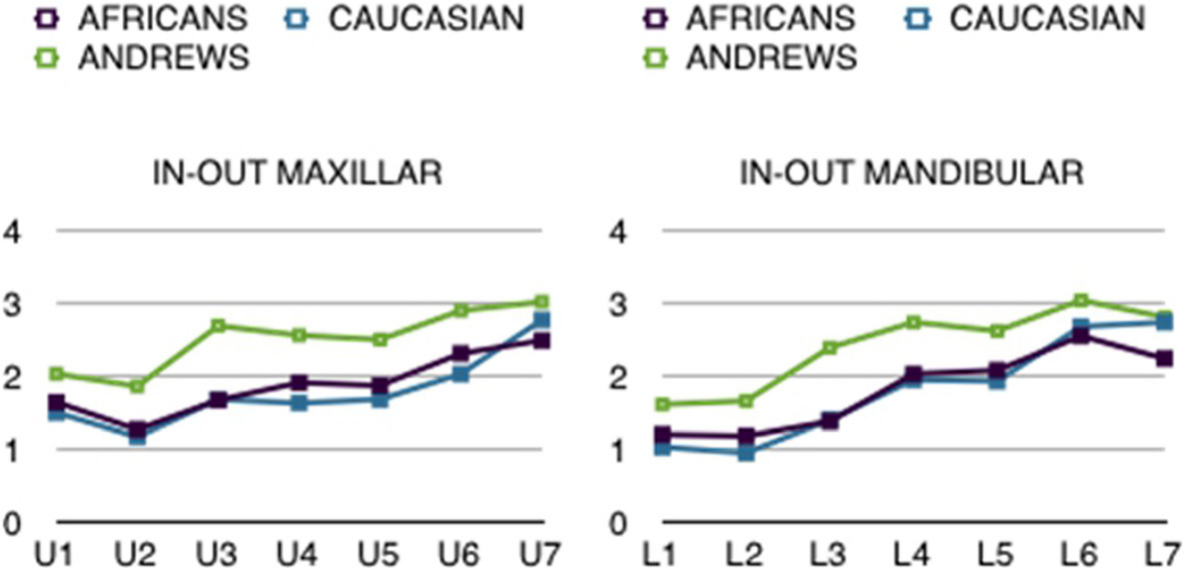
A comparison of in-out values we measured with those of Andrews

As regards the in-out measurements, those taken on our sample showed that the lateral incisors had the smallest values, whereas the molars were the most prominent. We also revealed a similar trend in both samples for the in-out values to progressively increase in an anteroposterior direction from the central incisor to the second molar. The African sample generally (incisors, premolars and first molars) presented greater in-out values than the Caucasian group in the upper jaw. In the lower jaw, the vestibular prominence of the tooth crowns was essentially similar in African and Caucasian subjects. With respect to Andrews’ values, however, we did notice a difference, as we revealed a tendency for the in-out values to increase towards the posterior sectors, where, according to the American author, there is none. Moreover, many of the values in our sample were smaller than those measured by Andrews, which may suggest that the coronal prominence in an anteroposterior direction is lower in Africans and Italian Caucasians than in North American Caucasians (Fig. 10).

Furthermore, the standard deviation values that emerged in our statistical analysis of the two samples were significant in some cases, thereby demonstrating that there is a considerable dispersion around the mean measurements for each tooth, especially in terms of torque. This finding, in agreement with previous studies [1–4, 6, 11–13], must be interpreted as a consequence of the biological variation in the inclination and angulation of the teeth, the variation in profile of the clinical crown, and variations in the inclination of the occlusal plane.

## Conclusion

- The measurement system used on our sample is repeatable.
- There is great dispersion around the mean values measured for each tooth (especially torque).
- Race and ethnicity greatly influence tip, torque and in-out values.
- Caucasians have more positive tip values and Africans, more positive torque values, with greater proclination of the incisors.
- In-out values were slightly greater in the African sample than in the Caucasian group, especially in the upper arch.
- Both Caucasian and African groups differ from Andrews’ sample in tip, torque and in-out values.
